# Epidemiologic and viral predictors of antiretroviral drug resistance among persons living with HIV in a large treatment program in Nigeria

**DOI:** 10.1186/s12981-020-0261-z

**Published:** 2020-02-17

**Authors:** Ernest Ekong, Nicaise Ndembi, Prosper Okonkwo, Patrick Dakum, John Idoko, Bolanle Banigbe, James Okuma, Patricia Agaba, William Blattner, Clement Adebamowo, Manhattan Charurat

**Affiliations:** 1 Department of Prevention, Care and Treatment, Institute of Human Virology-Nigeria (IHVN), Federal Capital Territory, Plot 252, Herbert Macaulay Way, Central Business District, Abuja, Nigeria; 2 Institute of Human Virology, University of Maryland School of Medicine, Baltimore, MD USA; 3 AIDS Prevention Initiative, Abuja, Nigeria; 4 Jos University Teaching Hospital, Jos, Nigeria

**Keywords:** Antiretroviral therapy, Acquired drug resistance, Predictors, HIV drug resistance testing, Low Middle Income Countries (LMICs), Resource-limited settings

## Abstract

**Background:**

Expanded access to combination antiretroviral therapy (cART) throughout sub-Saharan Africa over the last decade has remarkably improved the prognosis of persons living with HIV (PLWH). However, some PLWH experience virologic rebound after a period of viral suppression, usually followed by selection of drug resistant virus. Determining factors associated with drug resistance can inform patient management and healthcare policies, particularly in resource-limited settings where drug resistance testing is not routine.

**Methods:**

A case–control study was conducted using data captured from an electronic medical record in a large treatment program in Nigeria. Cases PLWH receiving cART who developed acquired drug resistance (ADR) and controls were those without ADR between 2004 and 2011. Each case was matched to up to 2 controls by sex, age, and education. Logistic regression was used estimate odds ratios (ORs) and 95% confidence intervals (CIs) for factors associated with ADR.

**Results:**

We evaluated 159 cases with ADR and 299 controls without ADR. In a multivariate model, factors associated with ADR included older age (OR = 2.35 [age 30–40 years 95% CI 1.29, 4.27], age 41 + years OR = 2.31 [95% CI 1.11, 4.84], compared to age 17–30), higher education level (secondary OR 2.14 [95% CI 1.1.11–4.13]), compared to primary and tertiary), non-adherence to care (OR = 2.48 [95% CI 1.50–4.00]), longer treatment duration (OR = 1.80 [95% CI 1.37–2.35]), lower CD4 count((OR = 0.95 [95% CI 0.95–0.97]) and higher viral load (OR = 1.97 [95% CI 1.44–2.54]).

**Conclusions:**

Understanding these predictors may guide programs in developing interventions to identify patients at risk of developing ADR and implementing prevention strategies.

## Introduction

Acquired and transmitted antiretroviral drug resistance mutations among persons living with HIV (PLWH) are a major public health concern, as they can limit the efficacy of available drugs for the management of HIV [1]. Resistance to antiretroviral (ARV) agents and subsequently increasing levels of transmitted, resistant virus have been identified by many researchers to potentially reverse the substantial gains achieved with potent ART [2–4]. Both transmitted drug resistance (TDR) and acquired drug resistance (ADR) reflect the relative usage of different ARV drugs in the population and the inherent genetic barrier to the development of resistance associated with individual drugs.

Development of drug resistance in chronic HIV infection has serious implications [5, 6]. Apart from the resultant limitations in the choice of effective treatment regimens, there are also extra cost implications associated with switches to second or third line therapy and extra demands on laboratory monitoring of patients [7–9]. Transmitted or pretreatment HIV drug resistance has significant impact on effectiveness of antiretroviral therapy. It leads to limitations in choice of effective regimen, longer time to achieve viral suppression and shorter time to virologic failure as compared to infection with a viral strain that is not drug resistant [10–12]. Even though several studies have shown that ADR is an independent predictor of virologic failure in naïve and treated HIV patients, factors that predict ADR are still not properly documented. Several studies suggest a 6% to 16% prevalence of HIV drug resistance in ART-naive patients [12, 13]. Virologic success has been shown to be predicted by high potency of ARV regimen, excellent adherence to treatment regimen [14], low viremia at ART initiation, higher CD4 count at ART initiation (> 200 cells/mm^3^) [15] and rapid reduction of viremia in response to treatment [16].

It is important to understand the interplay of factors associated with HIV drug resistance, especially in low to middle-income countries (LMICs) where there is limited access to viral load testing [17]. In this study we used data collected from the AIDS Prevention Initiative in Nigeria (APIN) program, a comprehensive HIV care and treatment program in the country, to evaluate sociodemographic, socioeconomic and other factors that could be associated or predict ADR in Nigeria.

## Materials and methods

### Study setting and study design

Since 2004, the AIDS Prevention Initiative in Nigeria (APIN) has provided care and treatment to more than 200,000 HIV/AIDS patients in several Nigerian cities, including: Lagos (Southwest zone, Lagos state), Jos (Northcentral zone, Plateau state), Ibadan (Southwest zone, Oyo state), and Maiduguri (Northeast zone, Borno state). This was a retrospective multi-centre case–control study of participants failing first-line ART at five Nigerian ART centers providing treatment and care (University College Hospital, Ibadan; National Institute for Medical Research, Lagos; Jos University Teaching Hospital, Jos; University of Maiduguri Teaching Hospital; and AIDS Prevention Initiative in Nigeria (APIN)). First-line treatment consisted of two nucleoside reverse transcriptase inhibitors (NRTIs), most commonly stavudine (d4T), zidovudine (AZT), or tenofovir (TDF), plus lamivudine (3CT) or emtricitabine (FTC) and one nonnucleoside reverse transcriptase inhibitor (NNRTI) such as nevirapine (NVP) or efavirenz (EFV). During the study period (2004–2011) we had three different WHO guidelines in 2003, 2006 and 2010 [18–20]. The study population included PLWH aged 18 years and above with at least two documented clinic visits during the study period of June 2004 through 31 December 2011. Initiation of first-line ART as defined by the national guidelines of the period included advanced immunodeficiency as defined by CD4 count, or advanced disease according to the World Health Organization (WHO) clinical stages. Patients with documented evidence of drug resistance (from genotypic drug resistance tests) between 2004 and 2011 from selected APIN sites were defined as cases, while those that had no evidence of antiretroviral drug resistance were the controls. Patients who did not start ART or require HIV therapy based on national treatment guidelines in effect at the time, patients without time of ART initiation electronic medical records, patients who made only a one-time HIV consultation visit, or those with less than 12 months of follow-up data, were excluded from the study.

### Data collection procedure and data collectors

The APIN electronic medical record system has been used in Nigeria since 2004. The system allows for longitudinal follow-up for all patients accessing care at the various treatment centers. Patients are given unique patient identifier numbers and tracked from program inception and during their follow-up visits. Patients’ demographic and clinical data routinely collected at outpatient counters, inpatient services, laboratory, voluntary HIV counseling and testing, and ART clinic and pharmacy, are linked for more efficient analysis of the prevention, care, and support program. Both case and control participants were initially identified from the dataset. Baseline demographic data at the time of ART initiation were recorded including sex, age, education, and occupation. Clinical data included CD4 cell count, HIV RNA level, TB and hepatitis status, date ART was commenced, current and past ART regimens, transfer of HIV care, resistance/mutations results, history of ARV experience, and the drug pickups, viral loads, and ARV drug history, were manually extracted from the individual patient’s case notes.

### Definitions

Measurement or determination of drug adherence was not standardized at the sites, with some using the pill-count, some pharmacy refill data, while others relied on self-report. In the pill count method, the unused pills were recorded for each patient on their subsequent clinic visit. The number of unused pills for the entire period of follow-up was obtained by totaling the pills unused in each month (cumulative unused pills). Where this information was available, adherence rate (%) was calculated as total doses taken as a percentage of total doses prescribed. CD4 cell count was measured every month during the first year and once every 3 months thereafter.

Treatment failure was categorized as virologic, immunologic or clinical. The definition of virologic failure evolved over time from two consecutive HIV ribonucleic acid (RNA) > 400 copies/ml after 24 weeks or > 50 copies/ml by 48 weeks in a treatment-naive patient or virological rebound (where there was a confirmed HIV-1 RNA > 50 copies/ml after initial virological suppression). Immunologic failure was defined as failure to achieve and maintain CD4 cell count > 350 cells/mm^3^ despite virological suppression (HIV-1 RNA < 50 copies/ml) after ≥ 2 years of antiretroviral treatment. For the purposes of this study, the WHO immunologic criteria for treatment failure used were a decline in the CD4 cell count to the value at ART initiation or below, a decline of at least 50% from the highest count on treatment or a persistent CD4 cell count below 100 cells/l after 6 months of ART [21]. Virologic failure was defined as a viral load of 1000 copies/ml (higher threshold) or as a viral load of 500 copies/ml (lower threshold) [21].

In clinical failure, there would have been the occurrence or recurrence of HIV-related events after at least 3 months of ART initiation, excluding immune reconstitution syndromes. At the beginning of program implementation, the most common first-line ART included stavudine (d4T), lamivudine (3TC), and nevirapine (NVP). In late 2006, the increased recognition of the toxicity and inferior efficacy of regimens containing d4T prompted the revision of international guidelines, with eventual removal of d4T from recommended first-line regimens. In 2008–2009, the introduction of generic tenofovir (TDF) equivalents and the fixed-dose combination (FDC) with emtricitabine (FTC) and efavirenz (EFV) further expanded usage of TDF in lieu of d4 [22].

### Statistical analysis

Using a test of proportion for difference between cases and controls (3% vs 13%), a two-sided type I error of 0.05, and power of 0.95, a minimum of 305 patients without ARV resistance and 153 patients with documented evidence of ARV resistance was required for the analysis. All statistical analyses were carried out using STATA version 11.0 (College Station, TX, USA) [23]. The total number of participants was 458 (299 without ADR and 159 with ADR).

We used mean and standard deviation (SD) to summarize continuous variables and proportions for categorical variables to describe basic characteristics of the study population. We tested for differences between cases and controls using the Chi square test for categorical variables and Wilcoxon Rank-sum test for continuous variables with p < 0.05 considered statistically significant. Logistic regression models were used to estimate Odd Ratios (OR) and 95% confidence intervals (CI). Univariate models were first run with ADR as the dependent variable and each of the predictors as independent variables. Factors that were associated with developing ADR at a p-value ≤ 0.20 in univariate models were further examined in multivariate models, with adjustment for a range of factors which included socio-demographic, clinical and immunologic characteristics, time of study enrolment and duration on ART. Final model included—potential confounders as well as established risk factors for developing ADR. Statistical analyses was conducted using SAS version 11.0 (SAS Institute, Cary, NC) statistical software.

## Results

A total of 458 study participants comprised of 299 (65.3%) HIV-infected individuals failing ART without ADR and 159 (34.7%) HIV-infected individuals failing ART with ADR were included in the analysis. The baseline (time to ART initiation) characteristics of the study participants is shown in Table 1. ARVDR+ and ARVDR− were significantly different by the proportion of married individuals (52.9% vs. 47.1%, p < 0.001), occupation (60% vs. 53%, p = 0.038), and education status (Secondary and Tertiary, 73% vs. 56%, p = 0.002). Mean duration on treatment was 3.5 years (SD = 1.3) for the ARVDR+ and 2.6 years (SD = 1.1) for the ARVDR− (p < 0.001). Mean CD4 count at ADR was 390 cells/µl (SD = 111 cells/µl) for ARVDR− and 170 cells/µl (SD = 72 cells/µl) for ARVDR+ (p < 0.001). Non-adherence to ART was higher among the ARVDR+ than the ARVDR−, 60% vs 29%, p < 0.001. There were no significant differences among ARVDR+ and ARVDR− for age, gender, baseline CD4 count and previous ART regimens (Table 1).

**Table 1:** Baseline characteristics of study participants

Patient characteristics	All patients n = 458	Without ADR N = 299	With ADR N = 159	P-value^a^
Socio-demographics				
Age (years), mean (SD)	34.7 (9.0)	34.3 (9.0)	35.5 (9.0)	0.08
Age categories (years) (%)				
17–30	163 (35.6%)	116 (38.8%)	47 (29.6%)	0.15
31–40	181 (39.5%)	112 (37.5%)	69 (43.4%)	
41 and above	114 (25.9%)	71 (23.8%)	43 (27.0%)	
Gender, female (%)	298 (65.1%)	200 (66.9%)	98 (61.6%)	0.26
Married status, married (%)	239 (52.9%)	177 (60.4%)	62 (39.0%)	< 0.001
Occupation (%)				
Unemployed	204 (44.5%)	141 (47.2%)	63 (39.6%)	0.038
Employed	254 (55.4%)	158 (52.8%)	96 (60.3%)	
Education (%)				
None	108 (23.6%)	80 (26.8%)	28 (17.6%)	0.002
Primary	67 (14.6%)	53 (17.7%)	14 (8.8%)	
Secondary	158 (34.5%)	93 (31.1%)	65 (40.9%)	
Tertiary	125 (27.3%)	73 (24.4%)	52 (32.7%)	
Clinical				
Year of enrolment (%)				
2004–2006	222 (58.9%)	125 (48.5%)	97 (80.2%)	< 0.001
2007–2011	157 (41.4%)	133 (51.6%)	24 (19.8%)	
ART treatment duration (years), mean (SD)	2.9 (1.2)	2.6 (1.1)	3.5 (1.3)	< 0.001
Baseline CD4 count (cells/µl), mean (SD)	160 (88)	161 (91)	159 (81)	0.98
Baseline CD4 count < 200 cells/µl (%)	277 (61.8%)	188 (62.9%)	89 (59.7%)	0.52
CD4 count at ADR (cells/µl), mean (SD)	317 (144)	390 (111)	170 (72)	< 0.001
CD4 count at ADR < 200 cells/µl (%)	109 (24.3%)	7 (2.3%)	102 (68.5%)	< 0.001
Baseline viral load log 10, mean (SD)	4.46 (1.00)	4.24 (1.02)	4.87 (0.84)	< 0.001
Previous ARV regimen (%)				
NNRTI	422 (92.1%)	278 (93.0%)	144 (90.6%)	0.36
PI	36 (7.9%)	21 (7.0%)	15 (9.4%)	
Non-adherence to care (%)	183 (40.0%)	87 (29.1%)	96 (60.4%)	< 0.001
Hepatitis B status (%)	97 (20.2%)	59 (19.7%)	38 (23.9%)	0.30

*NNRTI* non-nucleoside reverse transcriptase inhibitors, *PI* protease inhibitors, *ADR* adverse drug reaction, *ART* ARV therapy, *ARV* antiretroviral

^a^P-values from Chi squared-test for categorical variables or Wilcoxon test for continuous variables

In bivariate model, education (secondary and tertiary), year of enrolment, non-adherence, Hepatitis B status, treatment duration and baseline viral load were associated with the development of ADR. However, in a multivariate model, after adjusting for potential confounding variables, older age (age group 31–40 (OR = 2.35 [95% CI 1.29, 4.27], age group 41 + OR = 2.31 [95% CI 1.11, 4.84])), being unmarried (single) (OR = 0.40 [95% CI 0.24–0.67]), higher education level (secondary OR 2.14 [95% CI 1.1.11–4.13]; non-adherence to care (OR = 2.48 [95% CI 1.50–4.00]), longer treatment duration (OR = 1.80 [95% CI 1.37–2.35]), and higher viral load (OR = 1.97 [95% CI 1.44–2.54]) remained significantly associated with ADR (Table 2). Although the mean treatment duration overall was 2.9 years (SD = 1.2), those in the case group were longer on treatment (3.5 years, SD = 1.3) than those in the ARVDR− group (2.6 years, SD = 1.1). The study showed that for each year of treatment duration, the odds of developing ARVDR was higher (OR = 1.80, 95% CI 1.37 to 2.35, p < 0.001).

**Table 2:** Univariate and multivariate analyses of predictors of ADR

	%ADR n(%)	Univariate OR (95% CI), P-value	Multivariate OR (95% CI), P-value
Age			
17–30	47 (29.6%)	Reference	Reference
31–40	69 (43.4%)	1.52 (0.97–2.39), 0.07	2.35 (1.29–4.27), 0.005
41+	43 (27.0%)	1.50 (0.90–2.49), 0.12	2.31 (1.11–4.84), 0.026
Gender			
Female	98 (61.6%)	Reference	Reference
Male	61 (38.4%)	1.26 (0.84–1.88), 0.26	0.73 (0.42–1.29), 0.28
Marital status			
Single	97 (61.0%)	Reference	Reference
Married	62 (39.0%)	0.42 (0.28, 0.62), < 0.001	0.40 (0.24, 0.67), < 0.001
Occupation			
Un-employed	63 (39.6%)	Reference	Reference
Employed	96 (60.4%)	1.36(0.92–2.01), 0.12	1.17 (0.68–2.02), 0.56
Education			
None	28 (17.6%)	Reference	Reference
Primary	14 (8.8%)	0.76 (0.36–1.57), 0.45	0.61 (0.26–1.45), 0.26
Secondary	65 (40.9%)	2.00 (1.57–3.41), 0.011	2.14 (1.11–4.13), 0.02
Tertiary	52 (32.7%)	2.04 (1.17–3.56), 0.013	1.41 (0.69–2.85), 0.35
Year of enrolment			
2004–2006	97 (80.2%)	2.83 (1.88–4.26), < 0.001	1.11 (0.58–2.11), 0.76
2007–2011	24 (19.8%)	Reference	Reference
Enrolment year		0.51 (0.43–0.61), < 0.001	
Previous ARV regimen			
NNRTI	144 (90.6%)	0.73 (0.36–1.45), 0.36	
PI	15 (9.4%)	Reference	
Non-adherence			
No	63 (40.0%)	Reference	Reference
Yes	96 (60.4%)	3.71 (2.48–5.56), < 0.001	2.48 (1.50–4.00), < 0.001
Hepatitis B status			
No	121 (76.0%)	Reference	
Yes	38 (23.9%)	1.28 (0.80–2.03), 0.30	
Treatment duration (years)		1.93 (1.61–2.31), < 0.001	1.80 (1.37–2.35), < 0.001
Baseline CD4 count			
200+	60 (40.3%)	Reference	
< 200	89 (59.7%)	0.88 (0.59–1.31), 0.52	
CD4 count at ADR			
200+	47 (13.5%)	Reference	Reference
< 200	102 (68.5%)	0.97 (0.96–0.98), < 0.001	0.95 (0.94–0.97), < 0.001
Baseline viral load at ADR (log10)		2.08 (1.65–2.64), < 0.001	1.97 (1.44–2.54), < 0.001

*NNRTI* non-nucleoside reverse transcriptase inhibitors, *PI* protease inhibitors, *ADR* adverse drug reaction, *ART* ARV therapy, *ARV* antiretroviral

^a^P-values from Chi squared-test for categorical variables or Wilcoxon test for continuous variables

## Discussion

In this study we have shown that older age, being unmarried, duration of treatment > 2 years, non-adherence, low baseline CD4 count and high baseline VL seem to be associated with (predict of) ADR. These findings somewhat confirm, and at times diverge, from what has been described previously as factors associated with ADR. Although Khienprasit et al. [24] reported in a multivariate analysis that age < 40 years was predictive of ART failure, oOur findings indicate that older age PLWH are more likely to fail ART and switch to second line regimen, than younger patients. Our findings are in concordance with a large study conducted to assess the influence of age on immune recovery [25]. This effect of age on immune recovery with subsequent switch seems to be due to reduced thymic function that could impair immune recovery [26, 27]. Another reason for older patients to be more prone to ADR may be due to delayed diagnosis in this age group as HIV-associated symptoms can be mistaken for other diseases or even aging [28]. Older HIV patients are more susceptible to faster progression of the disease, with shorter and less symptomatic stage [28]. The use of other medications for concomitant co-morbidities among older patients may result in drug–drug interaction which predisposes to ADR and also predisposes to greater risk of opportunistic infections [29]. However, aging is generally expected to be a marker for greater maturity, lifestyle stability, and disease-specific education capable of affecting long-term adherence to therapy [30].

Married people usually have more family support so adherence to ART can be better handled or prevented through being reminded by the spouse or other family members. Marital status has been found to influence health and mortality, and give a lot of stability. Kiecolt-Glaser and Wilson [31] in their report on intimate partner relationships and health recorded that married people have significantly better health and a lower mortality than their single counterparts. Regarding HIV infection, social support has been linked to make for better adjustment [32], better treatment adherence [33, 34], and slower progression to AIDS [35, 36]. Molloy et al. [37] identified the presence of a primary partner as a key predictor of maintaining good health.

Another major predictor of ADR was education. However, when adjusted for confounders, only secondary education remained statistically significant. Two different studies, one in Southeast Nigeria, and another in Upper West Region of Ghana [38, 39] reported a negative association between education and adherence. The main reasons for this association could be the psychological state of the more educated person from stigmatization resulting in poor ART adherence. However, Rachlis et al. [40] have shown in a systematic review of studies in LMICs that higher education was associated with good adherence. A lower level of general education and poorer literacy may impact negatively on some patients’ ability to adhere, while a higher level of education has a positive impact [41].

The mean treatment duration was significantly different between those that developed ADR and those that did not. This result is not surprising. In the early part of the ART program in Nigeria there were months of drug stock-outs, lack of adequate supply management of medical commodities, weak laboratory infrastructure, and conditions that may favor the occurrence of high levels of ADR. Monitoring of treatment was only done by CD4 cell count and hardly viral load and genotype testing. Patients would therefore have been maintained on virologically failing regimens while multidrug resistant viruses accumulated and thereby made available drugs ineffective over time. In a comparable study in Tanzania by Asgeir et al. [42] the emergence of ADR in rural Tanzania was evaluated. Only a few studies have assessed long-term (> 2 years) emergence of drug resistance in sub-Saharan Africa. An early study from Senegal showed that 12.5% had one or more drug resistant mutations after a median of 30 months on ART (Laurent et al. [43]), whereas a study from Côte d’Ivoire found 22% resistance after a median period of 37 months on ART [44].

Incidentally, baseline CD4 cell count prior to ART initiation regarded as the most significant predictor of survival after initiation of first ART, was not strongly linked to the development of drug resistance. However, the CD4 count at development of ADR was very significant. The association between CD4 cell count and drug resistance has a biological reason. In this study, however, the analyses may have been partly confounded by the fact that most of the patients were enrolled on treatment with very low CD4 cell counts. Uy et al. [45] and Jose et al. [46] in their studies also separately reported that resistance occurs quite regularly in persons who initiate therapy later (with low CD4 count) during infection than in those who initiate ART much earlier. Earlier development of resistance may reduce available therapeutic options later [47]. The other significant observation found in this study was high viral load at ART initiation as a predictor of the development of ADRs in the future. This may be partly attributable to incomplete viral suppression in individuals with higher viral loads at ART initiation [48]. Ongoing low-level viremia is an independent risk factor for future viral failure. Another reason is due to the increased presence of drug-resistant minority HIV-1 variants in individuals with high viral load during untreated infection [49]. Nonadherence was shown to have a significant influence on the probability of ARVDR. O’Connor et al. [50] have previously demonstrated how prescription-refill data strongly predict CD4 cell decline, virologic response, and mortality after initiation of ART. Results from the present study confirm the association between adherence (estimated by prescription-refill percentages) and the development of drug resistance and provide insight into the way in which adherence influences therapy outcome. The results, however, differ from those of Bangsberg et al. [51] who reported that high levels of adherence (as high as 92–100%) do not prevent accumulation of drug-resistance mutations. In their study, it is likely that their subjects were enrolled in treatment at very high CD4 counts as well as being better prepared in adherence than those of our study. The importance of high adherence to antiretroviral therapy (ART) for HIV disease is well documented, and poor adherence can result in faster disease progression and ADR as well as increased health care costs and sickness., morbidity, mortality, and heightened risk of secondary HIV transmission [52].

The main limitation in this study was that data extracted from routine medical records may have been incomplete, inconclusive, or inaccurate. Additionally, it was difficult to know which factor acted first in the development of ADR in a patient who had several identified predictors. For the purposes of this study an association design was appropriate despite its limitations because the intention of this study was to determine if a relationship existed between the predictors and ADR.

The predictors outlined in this study should be recognised among vulnerable populations by health care providers especially in the resource-limited settings. It becomes critical to educate patients of possible onset of ADR especially those who are vulnerable based on these identified predictors. Policy makers, social advocacy groups and Health Ministries would use the information and be more focused in treatment and deploy resources to managing many more patients on first-line drugs instead of few on the much more expensive, scarce, second and third-line drugs due to the development of ADR.

## Data Availability

The data that support the findings of this study are PEPFAR data available from the APIN Public Health Initiatives Limited (APIN) but restrictions may apply to the availability of these data, which were used under license for the current study, and so are not publicly available. Data are however available from the authors upon reasonable request and with permission of APIN.
